# *RFC1* expansions are a common cause of idiopathic sensory neuropathy

**DOI:** 10.1093/brain/awab072

**Published:** 2021-05-09

**Authors:** Riccardo Currò, Alessandro Salvalaggio, Stefano Tozza, Chiara Gemelli, Natalia Dominik, Valentina Galassi Deforie, Francesca Magrinelli, Francesca Castellani, Elisa Vegezzi, Pietro Businaro, Ilaria Callegari, Anna Pichiecchio, Giuseppe Cosentino, Enrico Alfonsi, Enrico Marchioni, Silvia Colnaghi, Simone Gana, Enza Maria Valente, Cristina Tassorelli, Stephanie Efthymiou, Stefano Facchini, Aisling Carr, Matilde Laura, Alexander M Rossor, Hadi Manji, Michael P Lunn, Elena Pegoraro, Lucio Santoro, Marina Grandis, Emilia Bellone, Nicholas J Beauchamp, Marios Hadjivassiliou, Diego Kaski, Adolfo M Bronstein, Henry Houlden, Mary M Reilly, Paola Mandich, Angelo Schenone, Fiore Manganelli, Chiara Briani, Andrea Cortese

**Affiliations:** 1 Department of Brain and Behavioral Sciences, University of Pavia, Pavia, Italy; 2 IRCCS Mondino Foundation, Pavia, Italy; 3 Department of Neurosciences, ERN Neuromuscular Unit, University of Padova, Padova, Italy; 4 Department of Neuroscience and Reproductive and Odontostomatological Sciences, University of Naples Federico II, Naples, Italy; 5 Department of Neurosciences, Rehabilitation, Ophthalmology, Genetics, Maternal and Child Health (DINOGMI), University of Genoa, Genoa, Italy; 6 Neurology Unit, IRCCS San Martino Hospital, Genoa, Italy; 7 Department of Neuromuscular Diseases, UCL Queen Square Institute of Neurology, London, UK; 8 Department of Clinical and Movement Neurosciences, UCL Queen Square Institute of Neurology, London, UK; 9 Department of Neurosciences, Biomedicine and Movement Sciences, University of Verona, Verona, Italy; 10 Department of Molecular Medicine, Unit of Genetics, Università degli studi di Pavia, Pavia, Italy; 11 Medical Genetics Unit, IRCCS San Martino Hospital, Genoa, Italy; 12 Sheffield Diagnostic Genetics Service, Sheffield Children’s NHS Foundation Trust, Western Bank, Sheffield, UK; 13 Academic Department of Neurosciences, Sheffield Teaching Hospitals NHS Trust and University of Sheffield, Sheffield, UK; 14 Department of Brain Sciences, Neuro-otology Unit, Imperial College London, London, UK; 15 Department of Clinical and Motor Neurosciences, University College London, London, UK

**Keywords:** sensory neuropathy, chronic idiopathic axonal polyneuropathy, CANVAS, RFC1

## Abstract

After extensive evaluation, one-third of patients affected by polyneuropathy remain undiagnosed and are labelled as having chronic idiopathic axonal polyneuropathy, which refers to a sensory or sensory-motor, axonal, slowly progressive neuropathy of unknown origin. Since a sensory neuropathy/neuronopathy is identified in all patients with genetically confirmed *RFC1* cerebellar ataxia, neuropathy, vestibular areflexia syndrome, we speculated that *RFC1* expansions could underlie a fraction of idiopathic sensory neuropathies also diagnosed as chronic idiopathic axonal polyneuropathy. We retrospectively identified 225 patients diagnosed with chronic idiopathic axonal polyneuropathy (125 sensory neuropathy, 100 sensory-motor neuropathy) from our general neuropathy clinics in Italy and the UK. All patients underwent full neurological evaluation and a blood sample was collected for *RFC1* testing. Biallelic *RFC1* expansions were identified in 43 patients (34%) with sensory neuropathy and in none with sensory-motor neuropathy. Forty-two per cent of *RFC1*-positive patients had isolated sensory neuropathy or sensory neuropathy with chronic cough, while vestibular and/or cerebellar involvement, often subclinical, were identified at examination in 58%. Although the sensory ganglia are the primary pathological target of the disease, the sensory impairment was typically worse distally and symmetric, while gait and limb ataxia were absent in two-thirds of the cases. Sensory amplitudes were either globally absent (26%) or reduced in a length-dependent (30%) or non-length dependent pattern (44%). A quarter of *RFC1*-positive patients had previously received an alternative diagnosis, including Sjögren’s syndrome, sensory chronic inflammatory demyelinating polyneuropathy and paraneoplastic neuropathy, while three cases had been treated with immune therapies.

## Teaser

Currò et al. investigate the prevalence of RFC1 expansions in a cohort of patients with chronic idiopathic axonal polyneuropathy. RFC1 expansions were found in 34% of patients with sensory neuropathy, but in none with sensory-motor neuropathy. Clinical features and diagnostic clues that should lead to suspicion of RFC1 neuropathy are discussed.

See Roberts (doi:10.1093/brain/awab150) for a scientific commentary on this article.

## Introduction

Polyneuropathy is a common health problem leading to neurological consultation, with an estimated prevalence of 1–2.4% in the general population and up to 7% in people aged >65 years.^1^^,^^2^

Even after extensive evaluation, 25–32% of patients remain undiagnosed and are often labelled as having chronic idiopathic axonal polyneuropathy (CIAP).^3–6^ CIAP refers to a sensory or sensory-motor, axonal, slowly progressive neuropathy.^7^ Clinically, the disease burden falls predominantly or exclusively on the sensory fibres, especially at disease onset.^8^ The slow progression of CIAP with an accumulating burden of disability^9^ has raised the hypothesis of an underlying genetic cause.^10^ However, family history in CIAP is usually negative and age at onset is significantly higher than in most known inherited neuropathies.^9^^,^^11^ Previous attempts at identifying a genetic cause of CIAP were unsuccessful.^12^^,^^13^

Recently, biallelic intronic AAGGG repeat expansions in the replication factor complex subunit 1 (*RFC1*) gene were identified as the cause of cerebellar ataxia, neuropathy, vestibular areflexia syndrome (CANVAS) and a frequent cause of late onset ataxia.^14^^,^^15^ Notably, a sensory neuropathy/neuronopathy has been present in all the patients with biallelic *RFC1* expansions to date and can be observed as an isolated complaint in some, possibly reflecting an early stage of this complex neurological disease.^16^

Therefore, we speculated that biallelic *RFC1* expansions could account for a proportion of idiopathic sensory neuropathies also diagnosed as CIAP.

## Materials and methods

### Patient selection and clinical evaluation

**Figure 1:**
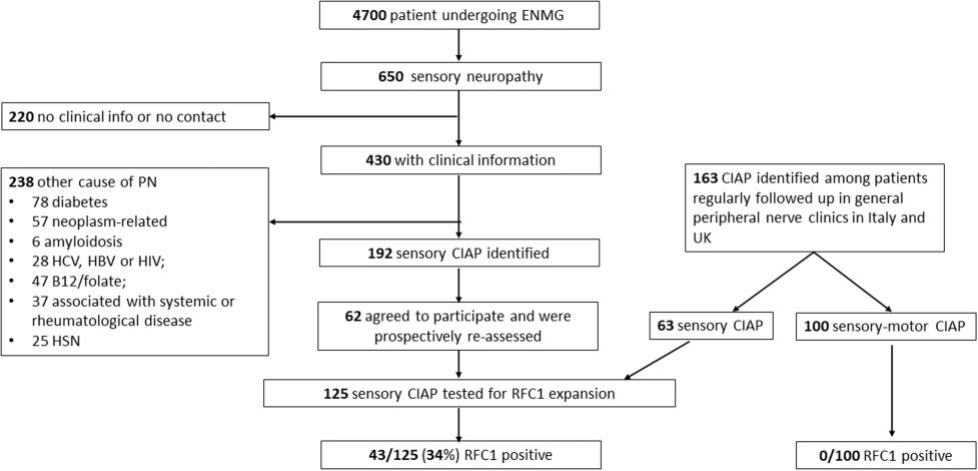
**Flow chart for enrolment of patients with sensory and sensory-motor CIAP.** ENMG = electroneuromyography; HSN = hereditary sensory neuropathy; PN = polyneuropathy.

Patients affected by CIAP were identified through the interrogation of clinical and neurophysiological databases from 2010 to 2019 in general peripheral nerve clinics in multiple centres in Italy and the UK. Patients were classified as having a sensory-motor or sensory neuropathy. Importantly, as sporadic CIAP is the focus of the study, patients previously diagnosed with CANVAS or with a family history of CANVAS were not included. The following laboratory investigations were performed in all cases: fasting blood glucose, glycosylated haemoglobin, folate, vitamin B12 and serum protein immunofixation electrophoresis.^12^ Depending on the clinical features, additional tests were performed, including those for markers of systemic autoimmunity (antinuclear antibodies, extractable nuclear antigens and antineutrophil cytoplasmic antibodies), paraneoplastic disorders (onconeural antibodies), HIV, hepatitis B and C infection, dosage of vitamin E, genetic testing for familial amyloid polyneuropathy and/or hereditary sensory neuropathies, fat pad aspiration for amyloid staining, imaging and nerve biopsy. A detailed algorithm for case enrolment is provided in Fig. 1.

Patients with sensory neuropathy (*n *=* *650) were assessed between 2010 and 2019 in two Italian University Hospitals (Pavia and Padova). Of these, 220 could not be contacted or had insufficient clinical information and were therefore excluded. Fifty-five per cent of 430 patients (*n *=* *238) were identified as having another cause of their sensory neuropathy, including diabetes (*n *=* *78), paraneoplastic syndrome (*n *=* *57), B12 and/or folate deficiency (*n *=* *47), neuropathy associated with systemic or rheumatological disease (*n *=* *37), genetic (*n *=* *25), acquired or familial amyloidosis (*n *=* *6) or hepatitis C, hepatitis B or HIV infection (n = 28) and were therefore excluded. We identified 192 patients with idiopathic sensory neuropathy and 62 agreed to take part in the study. These patients were clinically reassessed, and a DNA sample was obtained for *RFC1* testing. A further 163 patients with a diagnosis of CIAP were identified in general peripheral nerve clinics in Italy (Genova and Napoli) or the UK (London and Sheffield), including 63 patients with sensory neuropathy and 100 with sensory-motor neuropathy. Overall, 100 patients with sensory-motor and 125 with sensory CIAP were included in the study and tested for biallelic *RFC1* expansions.

### *RFC1* testing

The presence of *RFC1* expansions was assessed as previously described.^14^ Briefly, DNA was tested by flanking PCR and repeat-primed PCR for AAGGG repeat expansions in *RFC1*. Samples without amplifiable products on flanking PCR and a positive repeat-primed PCR for the AAGGG repeat were tested by Southern blotting in order to confirm the presence and measure the size of the biallelic *RFC1* expansions. Non-pathogenic AAAAG or AAAGG expansions were excluded by repeat-primed PCR in all the cases with positive repeat-primed PCR for AAGGG expansion.

### Ethics

The study was approved by the ethics committee of IRCCS Fondazione Policlinico San Matteo (p-20170028026) and by local institutional review boards. All patients gave informed consent prior to their inclusion in the study. The study complied with all relevant ethical regulations.

### Statistical analysis

Continuous data were expressed as medians (minimum-maximum). Statistical differences between subgroups of patients positive versus negative for *RFC1* expansions were tested using the Mann-Whitney test for quantitative data and Pearson’s chi-squared test for categorical data. All analyses were performed using STATA statistical software, version 14.

### Data availability

Anonymized data from this study will be shared by request from any qualified investigator.

## Results

### Genetic testing

Of 125 patients with sensory neuropathy 43 (34%) carried biallelic AAGGG repeat expansions in *RFC1*. Conversely, none of 100 axonal sensory-motor neuropathies had biallelic AAGGG expansions.

In *RFC1*-positive cases, the expansions size, as measured by Southern blotting, ranged from 249 to 2386 repeat units, with a median size of 661 repeats for the smaller allele and 810 for the larger allele (Supplementary Fig. 1).

### Clinical features of *RFC1*-positive patients

#### Symptoms

The demographic data and clinical features are detailed in Table 1. The median age at disease onset was 56 years (range: 30–75). Twenty-five patients were male and 18 were female. The family history for neuropathy was unremarkable in all cases apart from a patient with a sibling who had a diagnosis of sensory neuropathy.

**Table 1:** Clinical features of *RFC1*+ patients at disease onset and at most recent evaluation

Demographics	*n *=* *43	
Males	25 (58%)	
Positive family history	1 (2%)	
Age at onset	56 (30–75)	
Age at examination	67 (41–86)	

**Symptoms**	**Disease onset**	**Most recent evaluation**
Numbness	21 (49%)	27 (63%)
Unsteadiness	19 (44%)	30 (70%)
Tingling/pins and needles	14 (33%)	20 (46%)
Pain	10 (23%)	17 (39%)
Distribution		
Length dependent	NA	20/29 (69%)
Non-length-dependent	NA	9/29 (31%)
Dysautonomia	2 (5%)	9 (21%)
Dysarthria/dysphagia	1 (2%)	10 (23%)
Oscillopsia	1 (2%)	4 (9%)
Chronic cough	NA	26/37 (70%)

NA = not available.

At onset, 98% of patients (*n = *42) had symptoms of sensory neuropathy, including distal numbness (*n = *21), tingling or pins and needles sensation (*n = *14), unsteadiness (*n = *19) and pain (*n = *10). The distribution of sensory involvement was length-dependent in 69% of cases (*n = *20/29). Symptoms of dysautonomia (e.g. orthostatic hypotension, erectile dysfunction or altered sweating) were infrequent at onset (*n = *2). Only two patients reported symptoms suggestive of cerebellar (e.g. slurred speech or difficulties in swallowing) or vestibular dysfunction (e.g. oscillopsia) at disease onset. When specifically asked, 70% of patients (*n = *26/37) reported a chronic, paroxysmal dry cough, which was often previously attributed to asthma or gastro-esophageal reflux. In an asymptomatic 41-year-old male, the sensory neuropathy was suspected because of reduced or absent reflexes tested during a routine medical visit for workplace health surveillance.

#### Previous diagnoses

In 26% (*n = *11) of genetically confirmed patients, the neuropathy was previously attributed to a different cause, including inflammatory (*n = *7; one vasculitis, three CIDP, one Sjögren’s syndrome, one post-infectious, one paraneoplastic), metabolic (*n = *3, B12 deficiency) or toxic (*n = *1, previous exposure to hexavalent chromium). Three patients received immunosuppressive therapy (i.e. steroids, intravenous immunoglobulins or mycophenolate) and three had B12 replacement therapy.

Patients were followed up for 4.7 years (range 0–16.7 years). Notably, at the most recent examination, 70% of patients (*n = *30) still had symptoms of isolated neuropathy and did not report any vestibular or cerebellar complaint.

#### Disability

The disease was slowly progressive in 81% of cases (*n = *35) and stable in 19% (*n = *8). Thirty-seven per cent of patients (*n = *16) lost independent walking: 10 required unilateral support, five bilateral support and one was wheelchair-dependent.

#### Neurological examination

**Figure 2:**
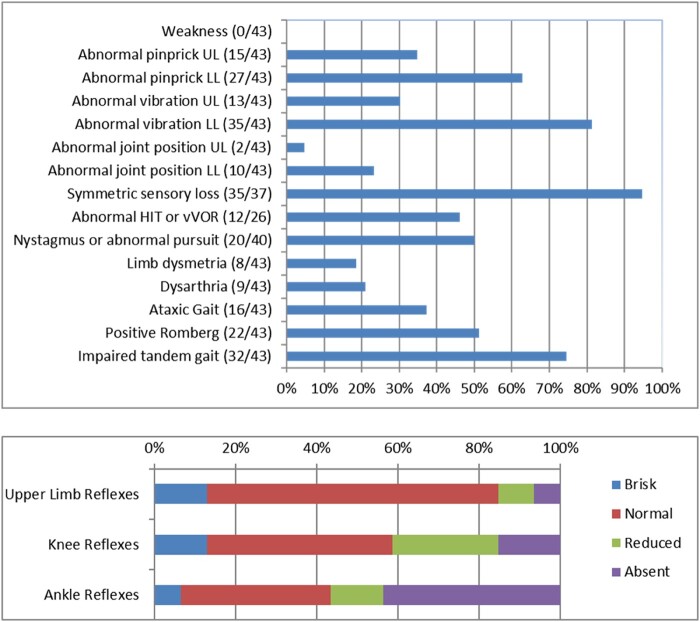
**Detailed neurological examination of *RFC1*+ patients.** HIT = head-impulse test; LL = lower limbs; UL = upper limbs; vVOR = visually-enhanced vestibulo-ocular reflex.

**Figure 3:**
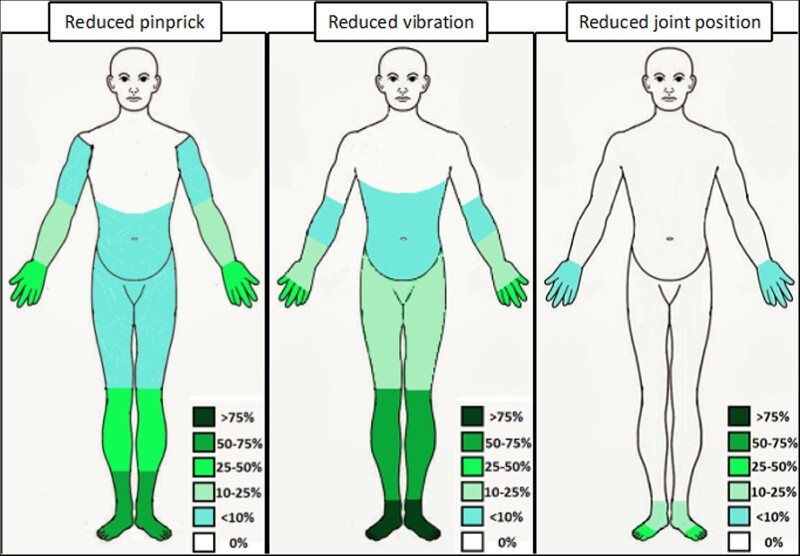
**Graphic representation of the distribution of abnormalities at sensory examination.** The different shades of colour correspond to different percentages of patients with reduced sensation in the specific area.

The data from the neurological examinations are summarized in Fig. 2. The median disease duration at the most recent examination was 9.6 years (range: 0.6–31.7 years). Gait was ataxic in 37% of patients (*n = *16), but difficulty in tandem walking was observed in 74% (*n = *32). Romberg was positive in 51% (*n = *22), supporting the presence of a prominent peripheral component of the unsteadiness. Eighty-six per cent of patients (*n = *37) had an abnormal sensory examination. Pinprick and vibratory sensation were impaired in 65% (*n = *28) and 81% (*n = *35) of patients, respectively. Conversely, joint position was impaired only in 23% of cases (*n = *10; Fig. 3). Sensory impairment was length-dependent (namely, lower limbs were more affected than upper limbs and distal limb segments were more affected than proximal) in 92% (*n = *34/37) and symmetric in 95% (*n = *35/37). Nineteen per cent (*n = *8) of patients showed incoordination during cerebellar testing, but the upper limbs were involved only in half of them. Strength, tone and plantar reflexes were normal in all patients. Reflexes were usually decreased or absent, but normal or brisk reflexes were observed in more than one-third of cases (*n = *11 and 5, respectively).

Abnormalities of eye movements were identified in 50% of cases, as revealed by the presence of nystagmus (*n = *19/40) or broken pursuit (*n = *9/34). Of 26 patients tested, 46% (*n = *12) had an altered bedside head-impulse test.

#### Nerve conduction studies

The detailed nerve conduction study results are reported in Table 2. The median delay from symptom onset to the first nerve conduction study was 5 years (range 0–31 years). Axonal loss of sensory fibres was generally severe, with globally absent sensory action potentials in 26% of cases (*n = *11), reduced in a length-dependent pattern (namely, a greater impairment in the lower than in the upper limbs) in 30% of patients (*n = *13) and reduced in a non-length dependent pattern in 44% (*n = *19). Sensory conduction velocities were normal or slightly decreased, consistently with a loss of fast-conducting large fibres, in all cases (median value of sural conduction velocity = 41.8 m/s, range: 31.4–51 m/s). We observed a clinical-electrodiagnostic dissociation with reduction in sensory amplitudes being worse than expected based on clinical findings in 44% (*n = *19) of cases: six patients with a normal sensory examination showed diffusely reduced sensory action potentials, with absent sensory action potentials throughout in two cases; 13 patients with reduced sensation restricted to distal segments of the lower limbs had reduced or absent sensory action potentials in the upper limbs in 38% (*n = *5) and 62% (*n = *8), respectively.

**Table 2:** Details of nerve conduction studies in *RFC1*+ patients

Sensory nerves	Reduced SAPs		Absent SAPs		Amplitude (µV)		SCV (m/s)
Reference Radial	– 10/21 (48%)		– 11/21 (52%)		>10 2.6 (0.37–9.4)		>48 50 (39–62)
Ref Ulnar	– 11/30 (37%)		– 18/30 (60%)		>6 2.4 (0.5–6.2)		>46 49.1 (39.7–61)
Reference Median	– 16/30 (53%)		– 14/30 (47%)		>8 3.4 (0.3–7.4)		>43.5 47 (36–56.5)
Reference Sural	– 18/39 (46%)		– 19/39 (49%)		>6 1.8 (0.2–11.6)		>42 41.8 (31.4–51)
Reference Sup. Peroneal	– 6/17 (35%)		– 11/17 (65%)		>6 1.8 (0.19–4)		>40 40 (37–43)

**Motor nerves**	**Reduced CMAPs**		**Absent CMAPs**		**Amplitude (mV)**		**MCV (m/s)**

Reference Ulnar	– 1/19 (5%)		– 0/19 (0%)		>5 9.6 (4.7–16.4)		>48 55 (50–73)
Reference Median	– 2/25 (8%)		– 0/25 (0%)		>6 10.4 (4–16.3)		>46.8 54 (48–62.9)
Reference Peroneal	– 2/34 (6%)		– 0/34 (0%)		>2 5 (0.7–17.1)		>41 45.2 (37.3–52.8)
Reference Tibial	– 1/21 (4%)		– 0/21 (0%)		>5 8.1 (2.9–19)		>40.6 43 (38.8–51.2)
**Length-dependent neuropathy = 13 (30%)**							
Absent LL SAPs/Reduced UL SAPs = 5				LL SAPs < UL SAPs = 8			
**Non length-dependent neuropathy = 30 (70%)**							
Absent SAPs at four limbs = 11		Absent UL SAPs/ Reduced LL SAPs = 5		UL SAPs < LL SAPs = 9		Globally absent SAPs with at least one SAP preserved = 5	

Values are expressed as median (range) or *n* (%). CMAPs = compound muscle action potentials; LL = lower limbs; MCV = motor conduction velocity; SAPs = sensory action potentials; SCV = sensory conduction velocity; Sup. Peroneal = superficial peroneal; UL = upper limbs.

Motor nerve conduction studies were normal in all but two patients with decreased compound muscle action potentials in the lower limbs.

#### Additional investigations

Cerebellar atrophy was identified in 26% of patients who underwent brain MRI (*n = *7/27). Clinical examination was more sensitive in detecting cerebellar abnormalities than brain MRI: only 5 of 20 subjects with clinical evidence of cerebellar dysfunction at examination had definite cerebellar atrophy on brain MRI. Conversely, subclinical cerebellar atrophy was identified in only two cases without cerebellar symptoms or signs at clinical examination.

Six patients with an abnormal bedside head-impulse test underwent vestibular testing, which confirmed a bilateral vestibular areflexia in all of them. CSF analysis was performed in 13 patients to rule out an inflammatory cause of the neuropathy and was normal in all. Four patients underwent a sural nerve biopsy, showing in all cases a diffuse and severe reduction of myelinated fibre density with no signs of regeneration (Supplementary Fig. 2).

### *RFC1* spectrum disorder: clinical syndrome

**Figure 4:**
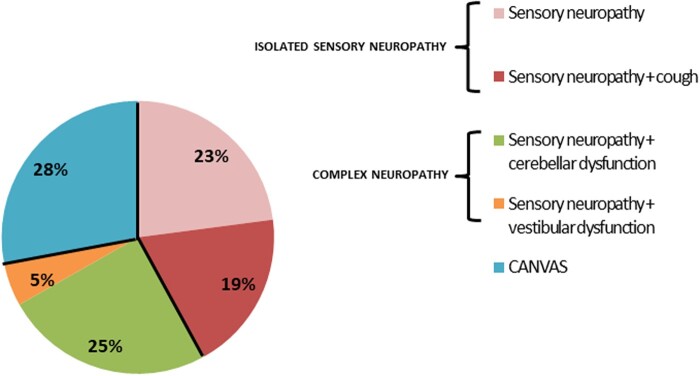
**Distribution of system involvement in *RFC1* patients after clinical examination and investigations.** Patients with isolated sensory neuropathy were further subdivided depending on the presence of cough; patients with complex neuropathy who did not have the full triad of CANVAS were further classified depending on the presence of vestibular or cerebellar dysfunction.

Based on history, neurological examination and ancillary investigations, after ∼10 years of disease duration, the clinical syndrome could be classified as isolated sensory neuropathy in 42% of patients (*n = *18), including 19% of cases with sensory neuropathy and cough (*n = *8), in 5% (*n = *2) sensory neuropathy with vestibular areflexia, in 25% (*n = *11) sensory neuropathy with cerebellar dysfunction and in 28% (*n = *12) full CANVAS (Fig. 4).

### Predictors of positive *RFC1* testing in patients with sensory neuropathy

To identify the symptoms and signs predictive of positive *RFC1* testing, we compared 43 cases with sensory neuropathy and biallelic AAGGG expansions with 58 cases also diagnosed with sensory neuropathy but negative for AAGGG repeat expansions (Table 3). The presence of cough, dysarthria and/or dysphagia, history of falls, clinical evidence of cerebellar or vestibular dysfunction at examination, reduced vibratory sensation above the knee in the lower limbs and a progressive course were more frequently observed in cases carrying biallelic *RFC1* expansions. Conversely, the two groups did not significantly differ in gender, age of onset, family history, report of vestibular symptoms, presence and distribution of sensory symptoms and signs (other than proximal reduction of vibratory sensation in the lower limbs), presence of gait and limb ataxia.

**Table 3:** Comparison of main clinical features and investigation results in *RFC1*-positive and -negative patients

	*RFC1*+	*RFC1−*	*P*-value
Mean age at onset	56 (30–75)	58 (12–85)	0.17
Male sex	25/43 (58%)	38/60 (63%)	0.59
Cough	26/37 (70%)	5/54 (9%)	**0.00**
Unsteadiness	30/43 (70%)	29/55 (53%)	0.08
History of falls	15/43 (35%)	6/56 (10%)	**0.00**
Chronic progression	35/43 (81%)	29/58 (50%)	**0.00**
Need for walking support	16/43 (37%)	6/58 (10%)	**0.00**
Sensory symptoms (non-length-dependent distribution)	9/29 (31%)	13/47 (28%)	0.95
Impaired vibratory sensation at knee or above	27/43 (63%)	13/57 (22%)	**0.00**
Non length-dependent pattern at NCS	30/43 (70%)	15/55 (27%)	**0.00**
Absent SAPs at UL	22/35 (63%)	10/40 (25%)	**0.00**
Cerebellar symptoms	10/43 (23%)	4/56 (7%)	**0.02**
Cerebellar signs	20/40 (50%)	12/58 (21%)	**0.00**
Vestibular symptoms	4/43 (9%)	1/58 (1%)	0.09
Vestibular signs	12/26 (46%)	2/37 (5%)	**0.00**

Values are provided mean (range) or *n* (%). Values in bold indicate significance. NCS = nerve conduction studies; SAPs = sensory action potentials; UL= upper limbs.

## Discussion

We identified *RFC1* AAGGG biallelic expansions in 34% of patients with sensory CIAP suggesting that *RFC1* expansions represent a significant genetic cause of sporadic sensory neuropathy.^12^^,^^17^

### Frequency of *RFC1* expansion in the general peripheral nerve clinic

The AAGGG allele frequency in the general population is 0.7–6.8%,^14^^,^^15^^,^^18^ resulting in an estimated prevalence at birth of biallelic carriers at risk of developing the disease ranging from 1 in ≃20 000 to 1 in ≃200 individuals. However, full CANVAS is considered relatively rare, while idiopathic sensory neuropathy is common in the ageing population.

We have previously hypothesized that sensory neurons could be the first system to be involved in a context of progressive disease leading to full CANVAS.^16^ This was based on the findings from a retrospective cohort of patients affected by late-onset ataxia, including familial cases, enrolled from highly specialized centres and with a specific expertise in neurogenetics and inherited neuropathies.^14^^,^^16^ Overall, the frequency of carriers of biallelic *RFC1* AAGGG expansions in that series was 22%. The frequency in sporadic sensory neuropathy has not yet been investigated.

In the present study, most of the cases were recruited from general peripheral nerve and electromyography clinics, without a bias towards genetic cases. Also, inclusion criteria were limited to the presence of sensory neuropathy and not of cerebellar ataxia or CANVAS. The study showed that in this population the frequency of *RFC1* expansion was as high as 34%, significantly higher than the frequency reported in previous ataxic cohorts, ranging from 2 to 22%.^14^^,^^18–21^ Thus, sensory nerve degeneration seems to play a major and early role in *RFC1*-related ataxic disorders.

**Figure 5:**
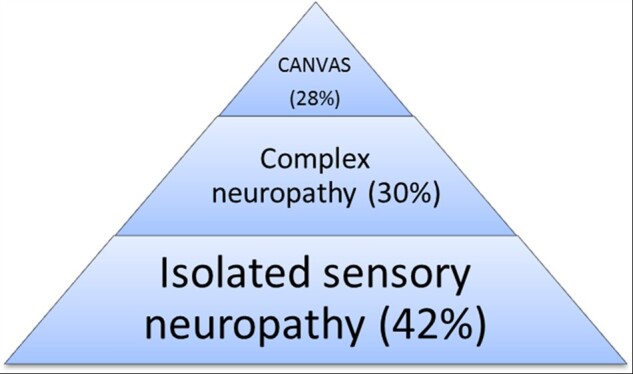
**The ‘iceberg’ hypothesis for RFC1 spectrum disorders.** Complex neuropathy was defined by the contemporary presence of neuropathy and vestibular or cerebellar involvement.

Although larger prospective studies are needed to elucidate the full spectrum and the natural history of RFC1 spectrum disorders, data from our cohort support an ‘iceberg’ hypothesis, where full CANVAS represents the tip, whereas isolated sensory neuropathy the bulk of the iceberg (Fig. 5).

### Clinical features and diagnostic clues of the *RFC1* disease spectrum

*RFC1*-positive cases typically reported complaints suggestive of distal symmetrical neuropathy including distal sensory loss, paraesthesia, pain. Interestingly, a clinical-neurophysiological dissociation between relatively mild clinical symptoms and signs, often in a length-dependent distribution, and a more severe and widespread alteration of sensory action potentials seems typical for *RFC1* sensory neuropathy and should prompt genetic testing.

Cerebellar symptoms were less common and oscillopsia rare. Although ataxia is a key component of the disease and the leading cause of disability during progression, unsteadiness was reported by less than half of cases at onset and observed in one-third at examination, while complaints of upper limb incoordination were rare (<10% of cases).

After clinical examination and investigation, the disease was confirmed as an isolated sensory neuropathy in 42% of *RFC1* cases after a median of 9.6 years of disease duration. Conversely, 58% had a complex neuropathy or CANVAS, usually based on the presence of subtle clinical signs, including nystagmus, broken pursuits and abnormal bedside head impulse test, which can be missed if not specifically looked for. The shorter disease duration in the present cohort compared to our previously published CANVAS cohort (9.6 years, range 0–31 years versus 20 years, range 0–50 years)^16^ may explain the lower percentage of complex disease and CANVAS and the lower degree of disability of our patients.

Interestingly, clinical examination seemed more accurate than brain MRI in detecting cerebellar involvement. Indeed, less than one-third of patients with signs of cerebellar dysfunction on examination had evidence of definite cerebellar atrophy on brain MRI, while vestibular testing was mostly confirmatory of abnormal bedside test. This is in accordance with previous studies, showing that investigations seldom show cerebellar atrophy and vestibular areflexia in patients with a normal neurological examination.^16^^,^^22^

### *RFC1* sensory neuropathy

The study also helped to better define the sensory neuropathy in carriers of biallelic AAGGG expansions in *RFC1*, including: (i) a length dependent distribution of sensory symptoms and signs in 69% and 92% of cases, respectively, mimicking a distal symmetrical polyneuropathy; (ii) the report of loss of sensation and positive sensory symptoms (which are unusual in genetic neuropathies) as the most common complaint at disease onset, and more frequent than unsteadiness; (iii) a preferential involvement of pinprick and vibration sense with relative sparing of position sense; (iv) variable reflexes, from absent to brisk (in accordance with previous studies showing a preservation of H and T-reflexes, as a result of the sparing of 1A muscle spindle afferent fibres)^23^^,^^24^; (v) a frequent clinical-neurophysiological dissociation between the length-dependent distribution of sensory symptoms and signs and the widespread impairment of sensory conduction; and (vi) the confirmation that the motor system is typically unaffected in *RFC1* disorder, as *RFC1* expansions were absent from 100 cases with sensory motor neuropathy and *RFC1*-positive patients showed no or minimal motor involvement.

### Chronic cough

The study also shows that a chronic cough is a strong diagnostic clue to suspect *RFC1* expansion in patients with a sensory neuropathy. Cough has been previously reported in patients affected by cerebellar ataxia^25^ and hereditary sensory neuropathy type 1B,^26^ but its origin remains unknown. In *RFC1* disease, cough often appears years or decades before the onset of sensory disturbances. Importantly, patients seldom spontaneously report cough as a symptom and need to be directly questioned about it. A history of asthma or gastroesophageal reflux should not cause this symptom to be dismissed in the context of a patient with sensory neuropathy.

### Misdiagnoses of *RFC1* neuropathy

Positive *RFC1* testing led to reclassification of 11 cases who had a previous alternative diagnosis, including sensory CIDP, Sjögren’s syndrome, paraneoplastic and metabolic neuropathy, and entailing three cases who had received unnecessary immune treatments. Therefore, it is of the utmost importance to consider *RFC1* in these cases to better inform patients on their condition and prognosis, to offer genetic counselling to patients and their families and to prevent therapeutic attempts with ineffective and possibly detrimental drugs.

## Conclusion

After the exclusion of the common acquired causes, *RFC1* should be considered in the diagnostic algorithm of all patients with sporadic sensory, but not sensory-motor, CIAP assessed in a general peripheral nerve clinic. Early diagnosis of patients carrying biallelic *RFC1* expansions, when the sensory neuropathy is still isolated, is crucial both for ensuring optimal patient management and for tracking the full natural history of the disease, including its early stages, when the neurodegenerative processes will likely be more amenable to therapeutic interventions.^27^

## Funding

A.C. thanks the Medical Research Council (MR/T001712/1), the Fondazione CARIPLO (2019-1836), the Italian Ministry of Health Ricerca Corrente 2018–2019, the Inherited Neuropathy Consortium (INC) and Fondazione Regionale per la Ricerca Biomedica for grant support. H.H. and M.M.R. thank the MRC, the Wellcome Trust, the MDA, MD UK, Ataxia UK, The MSA Trust, the Rosetrees Trust and the NIHR UCLH BRC for grant support. F.M. is supported by the European Academy of Neurology (EAN) Research Fellowship 2020.

## Competing interests

The authors report no competing interests.

## Supplementary material

Supplementary material is available at *Brain* online.

## Supplementary Material

awab072_Supplementary_Data

## Abbreviations

CANVAS: cerebellar ataxia, neuropathy, vestibular areflexia syndrome
CIAP: chronic idiopathic axonal polyneuropathy
