# Supplementary material for: RFC1 expansions are a common cause of idiopathic sensory neuropathy
Source: Brain. 2021 May 9;144(5):1542–50. doi: 10.1093/brain/awab072 (PMC8262986; doi:10.1093/brain/awab072)
Supplement: awab072_Supplementary_Data [file awab072_supplementary_data.zip › awab072-suppl_data/brain-2020-02306-File010.pdf]

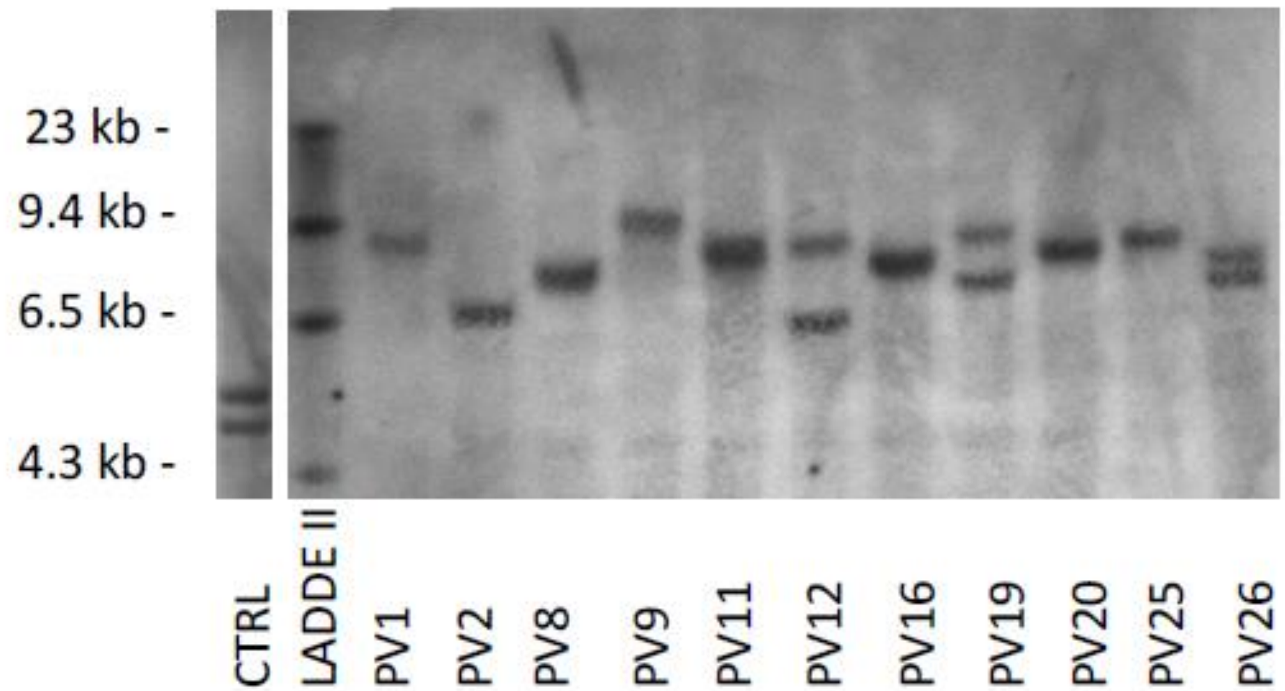


**Supplementary Figure 1. Southern blotting.** Representative examples of Southern blotting of genomic DNA from patients carrying biallelic AAGGG expansion in RFC1 (PV1, PV2, PV8, PV9, PV11, PV12, PV16, PV10, PV25, PV26) and a control (CTRL) with (AAAAG)11/.(AAAAG)exp haplotype. Patients show two discrete or overlapping bands ranging from 6.5 to 10 kb. Ladder used is DIG-labelled DNA Molecular Weight Marker II (Roche) (LADDER II) containing 8 fragments with the following base pair lengths: 125, 564, 2027, 2322 (not shown), 4361, 6557, 9416, and 23130 base pairs.
