# Supplementary material for: RFC1 expansions are a common cause of idiopathic sensory neuropathy
Source: Brain. 2021 May 9;144(5):1542–50. doi: 10.1093/brain/awab072 (PMC8262986; doi:10.1093/brain/awab072)
Supplement: awab072_Supplementary_Data [file awab072_supplementary_data.zip › awab072-suppl_data/brain-2020-02306-File011.pdf]

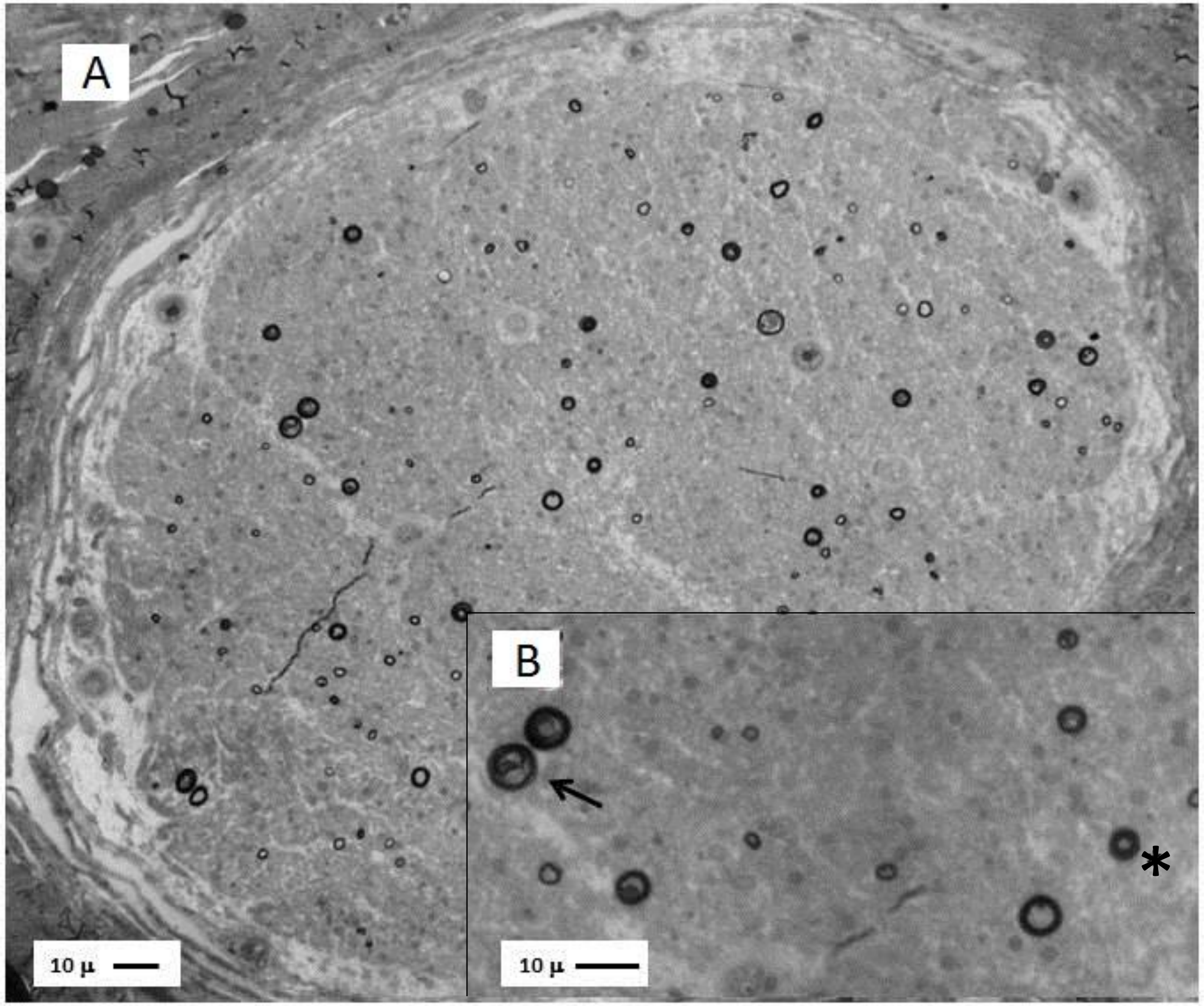

Supplementary Figure 2. Sural nerve biopsy. Semi-thin section stained with toluidine blue. A: the nerve biopsy shows diffuse and severe reduction of myelinated fibers density, with no signs of regeneration; B: at higher magnification, fibers undergoing early Wallerian degeneration (arrow) or showing axonal atrophy (asterisk) may be seen.
